# Supplementary figures and images for: PI Kinase-EhGEF2-EhRho5 axis contributes to LPA stimulated macropinocytosis in Entamoeba histolytica
Source: PLoS Pathog. 2022 May 20;18(5):e1010550. doi: 10.1371/journal.ppat.1010550 (PMC9173640; doi:10.1371/journal.ppat.1010550)

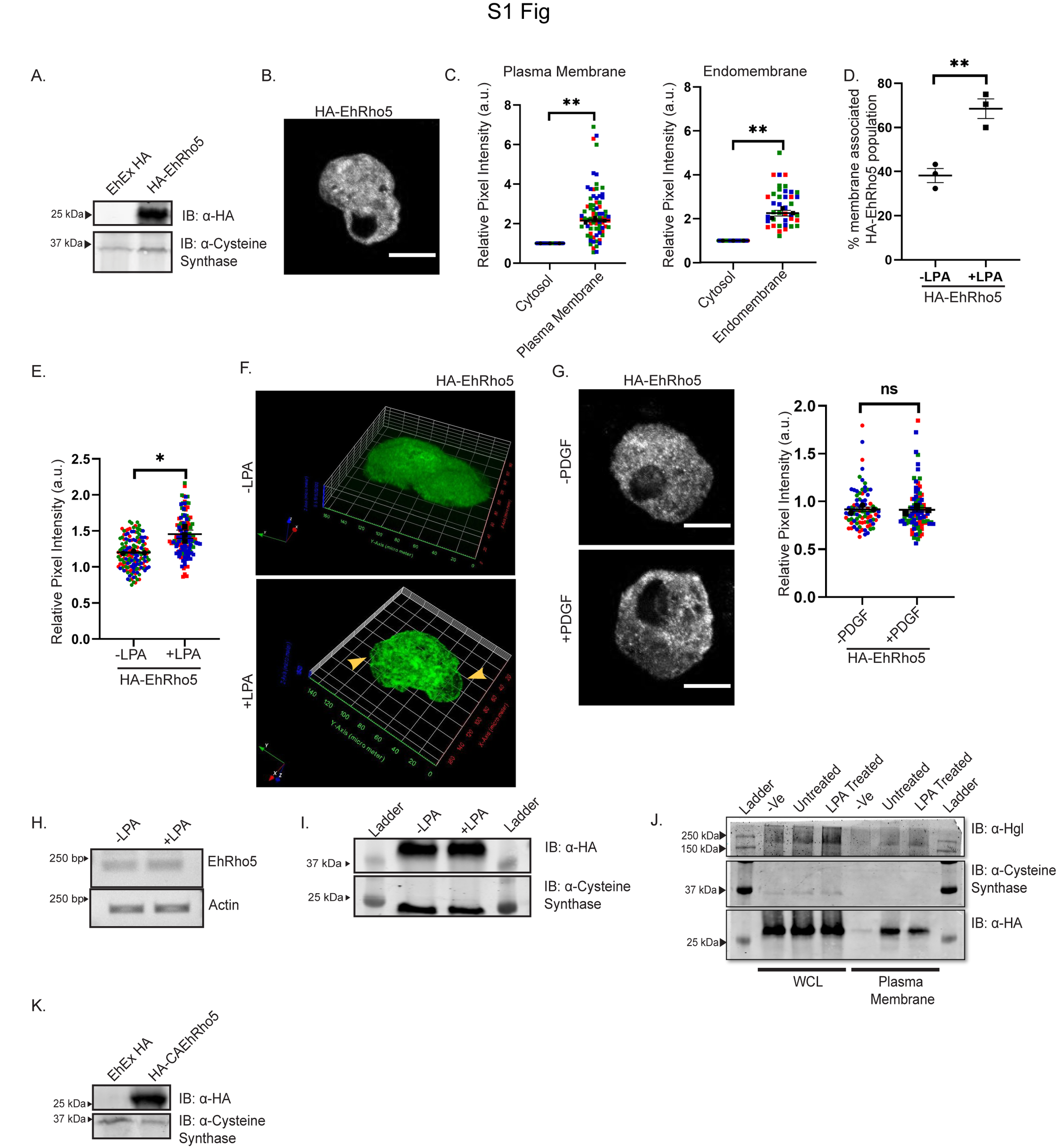

Supplement: S1 Fig — (A) 200μg cell lysates from plasmid EhEx HA (control) and HA-EhRho5 trophozoites were resolved on SDS-PAGE and subjected to immunoblotting using anti-HA and anti-cysteine synthase antibodies. Cysteine synthase was used as loading control. (B) Immunofluorescence image of HA-EhRho5 trophozoites, using anti-HA antibody (Scale bar = 10μm). (C) Relative pixel intensities are plotted as SuperPlots for HA-EhRho5 trophozoites in presence of LPA at plasma membrane and endomembrane. Normalisation was performed with EhRho5 cytosolic intensity. Statistical significance was determined using unpaired Student’s t-test (N = 3, n>80 (Plasma membrane), n≥40 (Endomembrane); **p<0.01). (D) A new cell line expressing HA-EhRho5 trophozoites was examined for LPA stimulation associated changes. Dot plot shows the percentage of cell population exhibiting membrane associated EhRho5 in presence as well as in absence of LPA, in HA-EhRho5 trophozoites. Values are represented as mean ± SEM of three independent experiments (N = 3, n≥80; unpaired Student’s t-test, **p<0.01). (E) Quantitative analysis of relative pixel intensities in LPA treated cells compared to untreated in new cell line expressing HA-EhRho5. SuperPlot shows comparison of relative pixel intensities of HA-EhRho5 fluorescence at plasma membrane in LPA treated and untreated cells (N = 3, n≥80; unpaired Student’s t-test, *p<0.05). (F) 3D reconstruction of HA-EhRho5 trophozoites, post stimulation with and without LPA. Arrowheads show the membrane regions. (G) HA-EhRho5 serum starved trophozoites were stimulated with 60ng/μl PDGF. Cells were fixed and immunostained using anti-HA antibodies (Scale bar = 10μm). Quantification of relative pixel intensity at plasma membrane in presence and in absence of PDGF. Values were plotted as mean ± SEM in SuperPlots. N = 3, n = 85, unpaired Student’s t-test, p>0.05. (H) Expression analysis of EhRho5 mRNA in presence and absence of LPA stimulation. Wild type trophozoites were stimulated with or witho [file ppat.1010550.s001.tif]

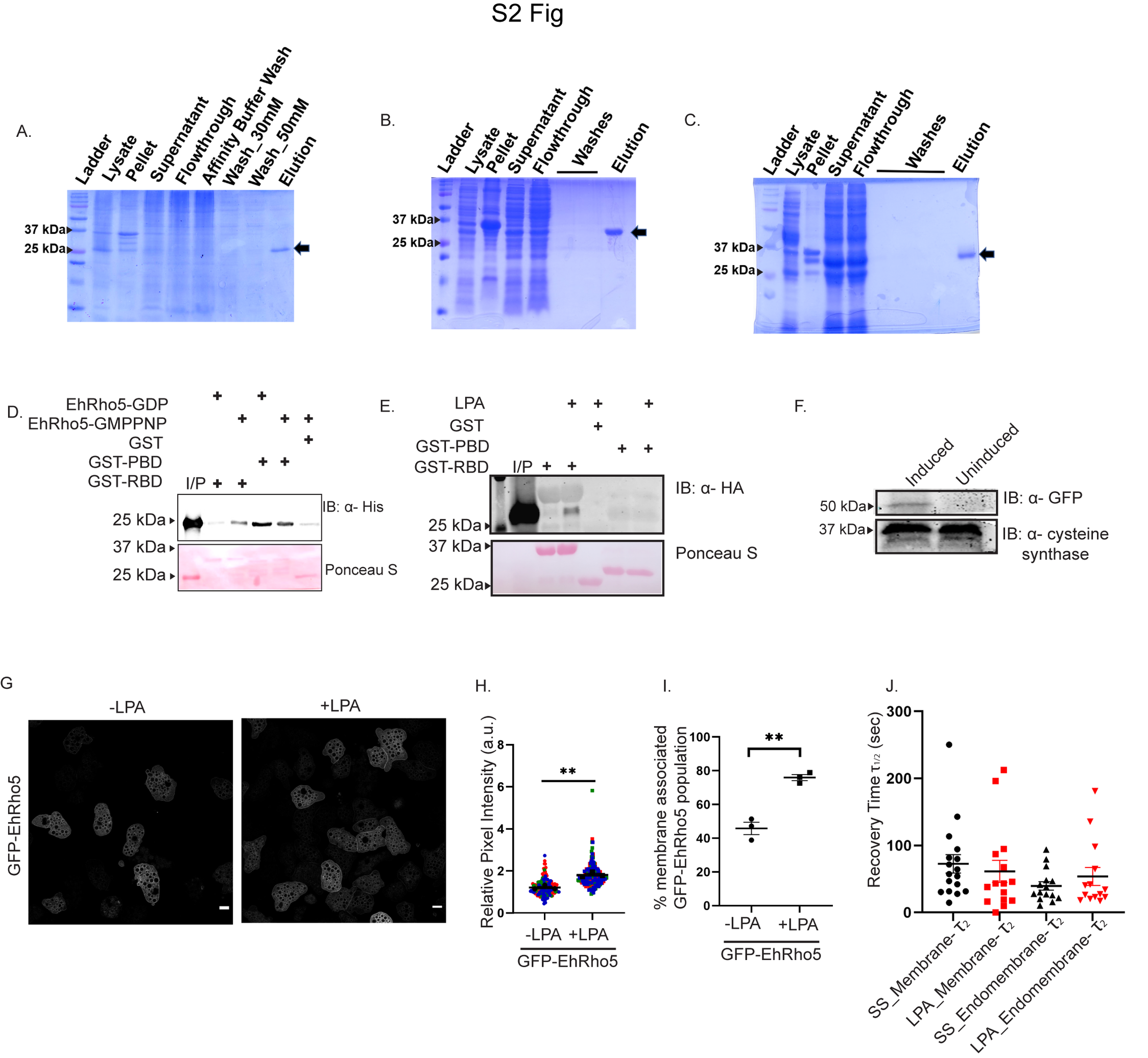

Supplement: S2 Fig — (A-C) Panel shows protein purification profiles of His-EhRho5, GST-RBD and GST-PBD respectively. Purified proteins are indicated with an arrow. (D) Additional immunoblot of In vitro binding assay (ref Fig 2A). (E) Additional immunoblot of Rho activation assay (ref Fig 2C). (F) GFP-EhRho5 expressing trophozoites induced with 30μg/ml tetracycline for 48 hrs were lysed in lysis buffer. 300μg cell lysates were resolved on SDS-PAGE and subjected to immunoblotting using anti-GFP and anti-CS antibodies. Cysteine synthase and uninduced lysate were used as control. (G) A new cell line expressing GFP-EhRho5 trophozoites was examined for LPA stimulation associated changes. Serum starved GFP-EhRho5 trophozoites were stimulated with LPA. Representative image shows the localisation of GFP-EhRho5 in presence as well as in absence of LPA (Scale bar = 10μm). (H) Quantitative analysis of LPA treated cells compared to untreated. SuperPlot shows comparison of relative pixel intensities of GFP-EhRho5 fluorescence at plasma membrane and endomembrane (N = 3, n≥150; unpaired Student’s t-test, **p<0.01). (I) Dot plot shows the percentage of cell population exhibiting membrane associated EhRho5 in presence as well as in absence of LPA, in GFP-EhRho5 trophozoites. Values are represented as mean ± SEM of three independent experiments (N = 3, n≥120; unpaired Student’s t-test, **p<0.01). (J) Representation of fluorescence recovery time τ2 for indicated conditions. (TIF) [file ppat.1010550.s002.tif]

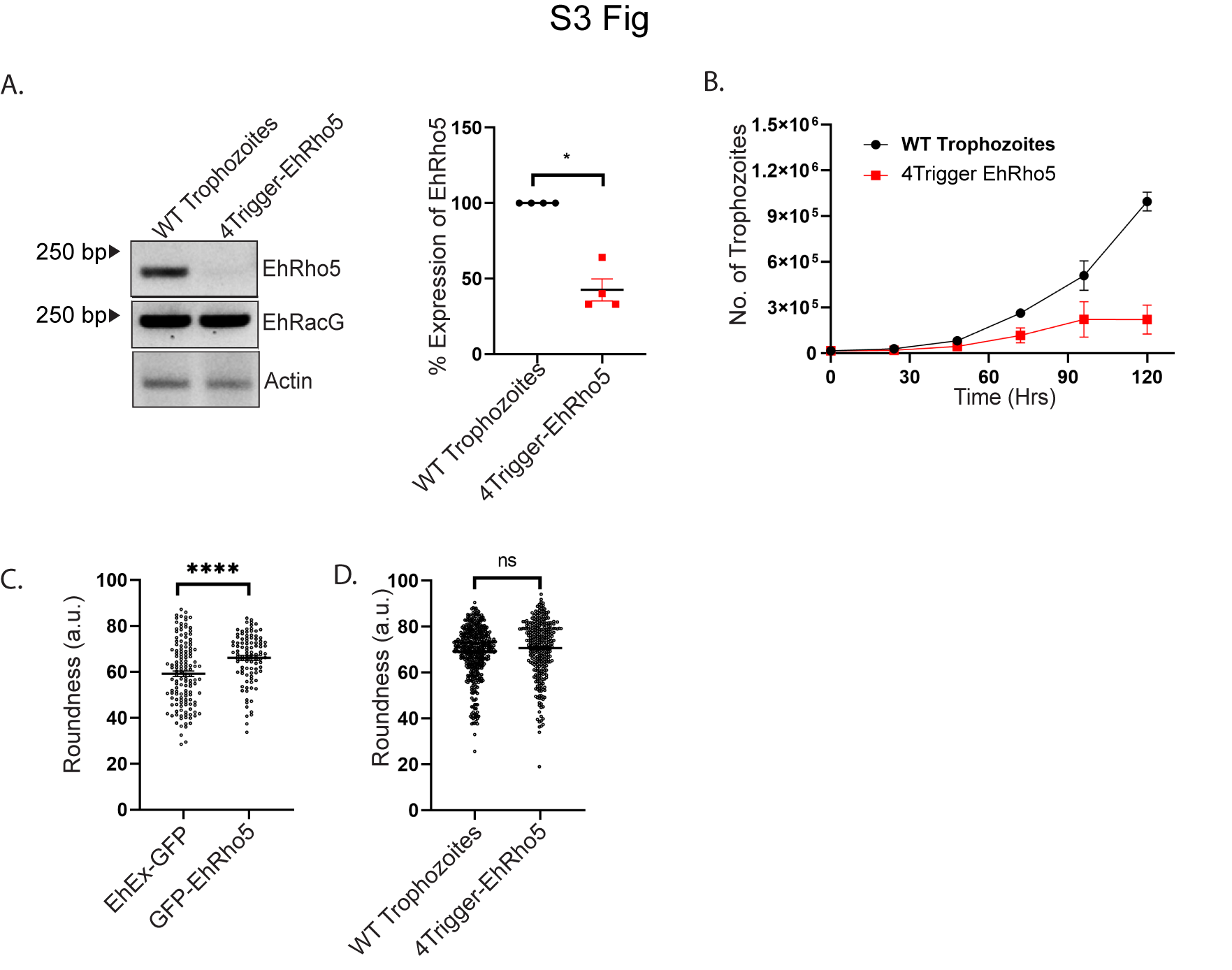

Supplement: S3 Fig — (A) Confirmation of Knockdown of EhRho5. The knockdown efficiency was determined by Semi-Q PCR. Normalisation of EhRho5 expression was done with actin band intensity (N = 4, mean ± SEM, ratio paired Student’s t-test, * p<0.05). EhRacG (EhRho2; 55% identity with EhRho5) was used as control to check the specificity of knockdown. (B) RNAi mediated knockdown of EhRho5 affects growth of trophozoites. Trophozoites were grown for 5 days and every 24hrs cells were harvested and counted. (C, D) Roundness of the cells was calculated using ICY software and plotted. (N = 3, n>140, unpaired Student’s t-test, ns (non-significant) p>0.05, ****<0.0001). (TIF) [file ppat.1010550.s003.tif]

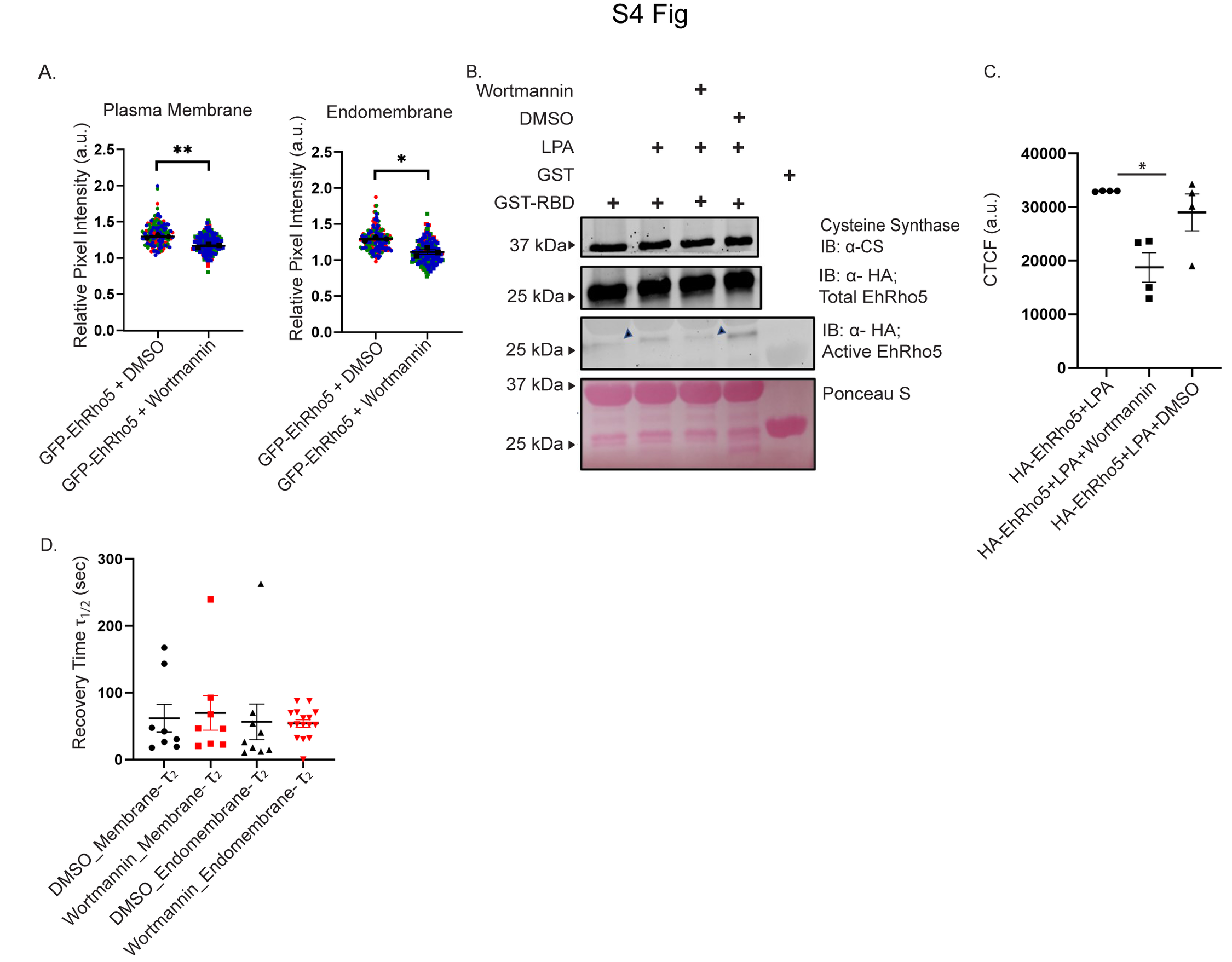

Supplement: S4 Fig — (A) Relative pixel intensities are plotted as SuperPlots for LPA pre-treated GFP-EhRho5 trophozoites upon wortmannin or DMSO treatment. N = 3, n>140, mean ± SEM, unpaired Student’s t-test, **p<0.01, *p<0.05) (B) Panel shows additional immunoblot (ref. to Fig 4C). (C) Serum starved LPA pre-treated HA-EhRho5 trophozoites were studied for their dextran uptake for indicated conditions. Trophozoites were treated with TR-Dextran along with wortmannin and DMSO, individually. Cells were fixed with 4% PFA and immunostained with anti-HA antibody, followed by image acquisition in a confocal microscope. CTCF was determined for each condition as described in Materials and Methods. Values represent means ± SEM, N = 4, n>60, ratio-paired Student’s t-test *p<0.05). (D) Representation of fluorescence recovery time τ2 for indicated conditions. (TIF) [file ppat.1010550.s004.tif]

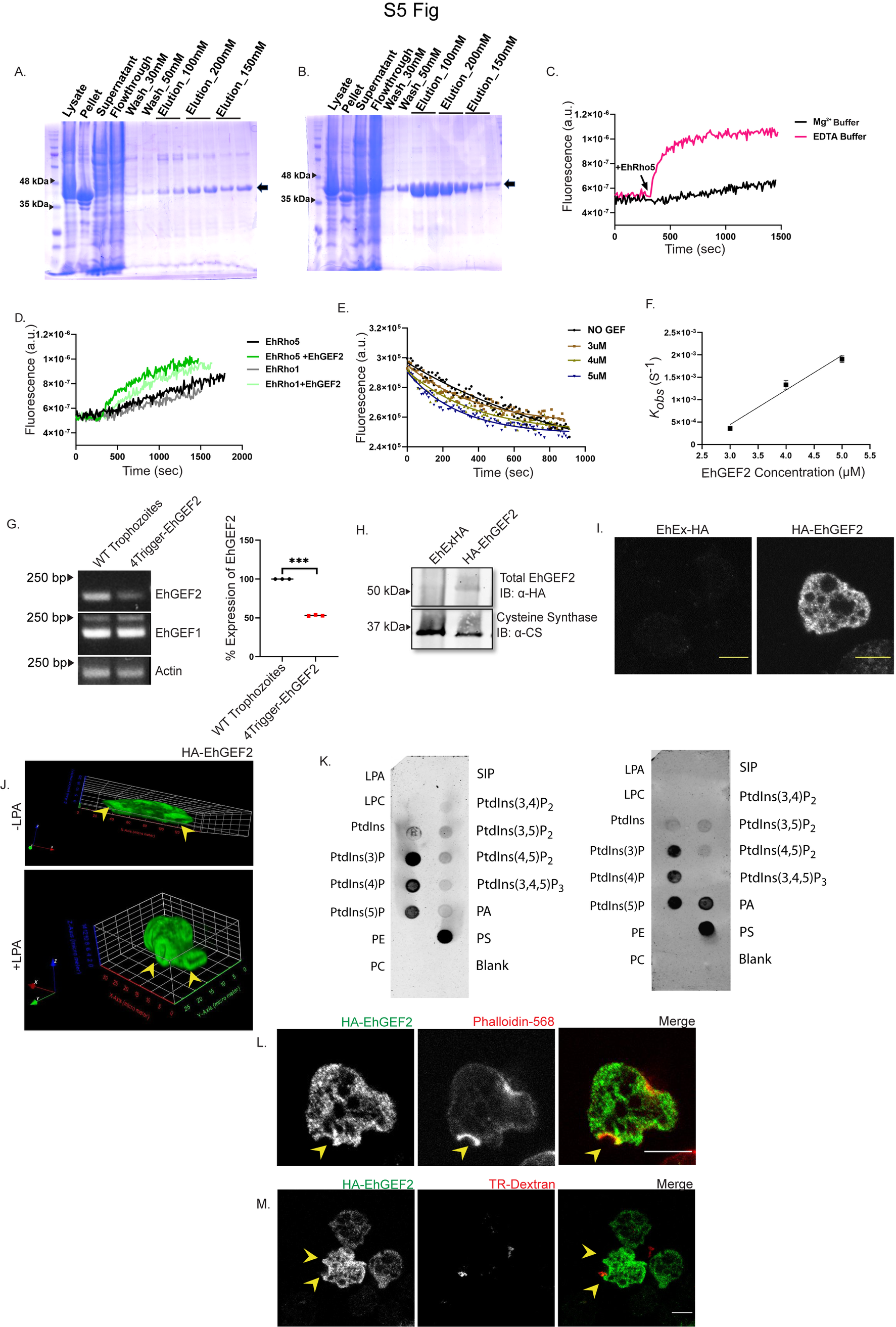

Supplement: S5 Fig — (A-B) Purified His-tagged EhFP4 and EhGEF2, respectively, were resolved with SDS-PAGE followed by staining with Coomassie Blue. Arrows indicate the affinity purified proteins. (C) In vitro functional analysis of EhRho5. Nucleotide exchange kinetics of EhRho5 (2μM) in Mg2+ and EDTA buffer. (D) Exchange kinetics of EhRho1 (2μM) and EhRho5 (2μM) in presence and in absence of EhGEF2 (1μM). A single replicate data trace is shown. (E) GEF activity exhibited by different concentrations of EhGEF2 for 2μM of EhRacG (EhRho2). Panel shows a single data trace. (F) Catalytic efficiency (kcat/km) was obtained from the slope of a linear least square fit of kobs values against EhGEF2 concentration. Values represent mean ± SEM of three independent measurements (N = 3). (G) Knockdown confirmation of EhGEF2. The knockdown efficiency was determined by Semi-Q PCR. EhGEF2 expression was normalised to actin (N = 3, ratio paired Student’s t-test, ***p<0.001). Specificity of knockdown was checked with expression of EhGEF1 (25% identity with EhGEF2) (H) 200μg cell lysate, each from EhEx HA (control) and HA-EhGEF2 trophozoites were resolved on SDS-PAGE and subjected to immunoblotting using anti-HA and anti-CS antibodies. (I) Representative images of HA-EhGEF2 overexpressing trophozoites compared to EhEx HA (control), scale bar = 10μm. (J) Representative 3D reconstructions of HA-EhGEF2 trophozoites, in presence or in absence of LPA (15μM). Arrowheads represent the macropinocytic cups. (K) Additional lipid blots show the binding of His-EhGEF2 to different phosphoinositides. (L) Confocal image exhibiting co-existence of HA-EhGEF2 and F-actin on macropinocytic cups, indicated by arrowheads (Scale bar = 10μm). (M) Representative confocal image of HA-EhGEF2 trophozoites performing TR-Dextran uptake. EhGEF2 is localised at the tips of macropinocytic cups, shown by arrowheads (Scale bar = 10μm). (TIF) [file ppat.1010550.s005.tif]

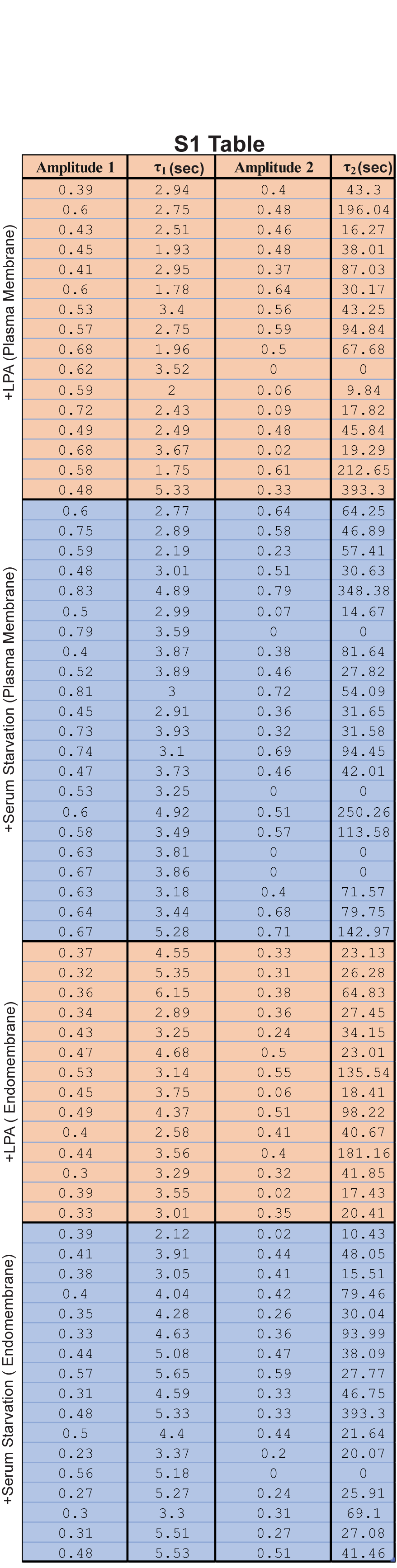

Supplement: S1 Table — (TIF) [file ppat.1010550.s006.tif]

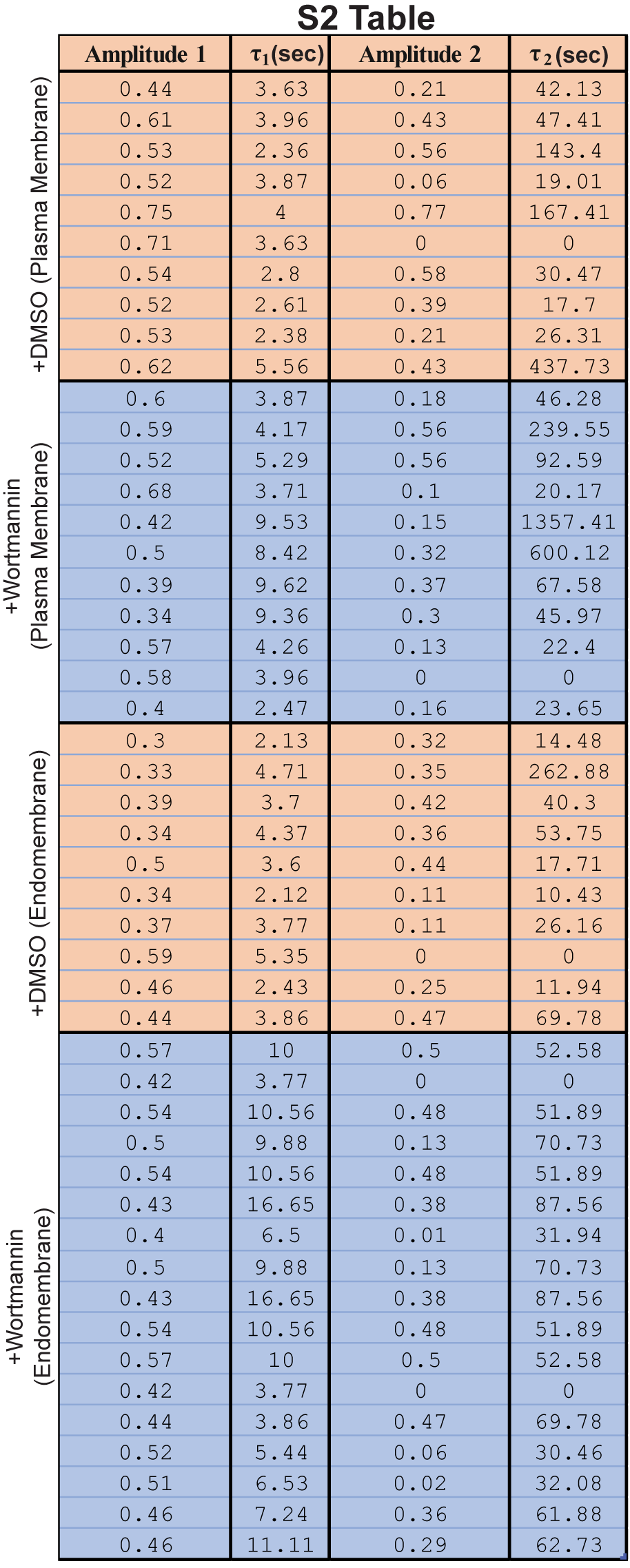

Supplement: S2 Table — (TIF) [file ppat.1010550.s007.tif]

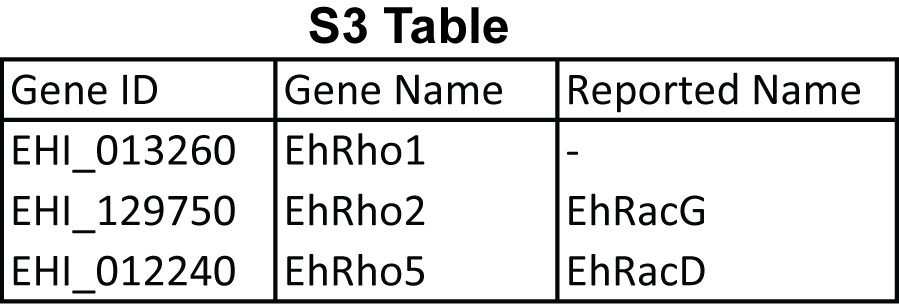

Supplement: S3 Table — (TIF) [file ppat.1010550.s008.tif]
