# Supplementary material for: PI Kinase-EhGEF2-EhRho5 axis contributes to LPA stimulated macropinocytosis in Entamoeba histolytica
Source: PLoS Pathog. 2022 May 20;18(5):e1010550. doi: 10.1371/journal.ppat.1010550 (PMC9173640; doi:10.1371/journal.ppat.1010550)
Supplement: Attachment — Submitted filename: Response to reviewers.docx [file ppat.1010550.s018.docx]

**Response to Reviewers**

**Part II – Major Issues: Key Experiments Required for Acceptance**
Please use this section to detail the key new experiments or modifications of existing experiments that should be absolutely required to validate study conclusions.

Generally, there should be no more than 3 such required experiments or major modifications for a "Major Revision" recommendation. If more than 3 experiments are necessary to validate the study conclusions, then you are encouraged to recommend "Reject".

Reviewer #1: (No Response)

Reviewer #2: The main question in this study is unclear. The molecular mechanism of signaling to stimulate macropinocytosis is an important and interesting topic to study, however, the importance of studying LPA stimulated macropinocytosis in this parasite is not fully explained. Furthermore, the authors stated macropinocytosis in this parasite is stimulated via the PI3K-GEF2-Rho5 axis without explaining the uniqueness and conservation of this signal in model organisms. What is the significance of LPA and its signaling in this parasite? How does this link to pathogenesis? Please clarify the reason why the authors believe LPA and PDGF mediated Rho activation signaling pathway exists in E. histolytica. What is the physiological state the authors hypothesized? Related to these points, the difference between LPA and PDGF did not explain. Please reconsider the story to discuss and clarify these points (Huston and Sateriale 2011)

Response: We thank the reviewer for pointing out the lack of justification for studying LPA mediated signalling in *Entamoeba histolytica*. We have now elaborated and discussed the suggestions which has enriched the manuscript. While transiting from intestinal site of infection to the extraintestinal secondary infection sites, such as in case of hepatic amoebiasis, amoebic trophozoites enters the bloodstream, where they experience various factors from the host environment and may utilize them for nutrient acquisition and pathogenesis. LPA and PDGF are among such few host factors, produced by platelets which are present abundantly in the bloodstream (Czarkowska-Paczek, Bartlomiejczyk, and Przybylski 2006; Sugiura et al. 2002) It is well established in cells of mammalian origin that LPA and PDGF selectively activate Rho and Rac subfamily respectively, to elicit downstream signalling (Van Aelst and D’Souza-Schorey 1997; Moolenaar et al. 1997). Earlier studies have shown the translocation of EhRho1 to vesicular membrane and to some extent to the plasma membrane post treatment with LPA. Also, activated (GTP bound) EhRho1 was detected in the amoebic lysates stimulated with LPA (Franco-Barraza et al. 2006; Bharadwaj et al. 2017). Similarly fibronectin has been implicated in regulating Rho family of GTPases and cytoskeleton in Entamoeba histolytica (Ríos et al. 2008). Various examples of induced signalling by Tumor Necrosis Factor, fibronectin, serum has been demonstrated in E. histolytica (Blazquez et al. 2006; Shrimal, Bhattacharya, and Bhattacharya 2010; Franco-Barraza et al. 2006; Blazquez et al. 2008; Bharadwaj et al. 2017). Involvement of PI3-Kinases in TNF and LPA induced signalling has been demonstrated in *Entamoeba histolytica*. LPA stimulated EhRho1 has been reported to regulate PIP3 levels via PI3Kinase and invasive behaviour of trophozoites, whereas TNF induced signalling led to PI3K dependent chemotaxis (Blazquez et al. 2008; Bharadwaj et al. 2017; Seastone et al. 1998). The discrete pieces of information from the published studies, prompted us to determine the signalling cascade and their connecting links. (Line 82-99)

For pathogenesis and nutrient acquisition within the host, cells perform macropinocytosis and phagocytosis, collectively known as heterophagy (Buckley 2018). Despite major differences in stimulus and size, formation of both macropinosomes and phagosomes share the same regulatory proteins. Phagocytosis in the amoebic trophozoites, particularly in initiation phase of formation has been investigated in detail (Babuta et al. 2015; Verma and Datta 2017; Bharadwaj et al. 2021; Mansuri, Bhattacharya, and Bhattacharya 2014; Verma, Nozaki, and Datta 2016), but studies to shed light on the mechanism of macropinocytosis are very much limited for the amoeba. This prompted us to investigate the signalling responsible for macropinocytosis. (line 42-48)

The nomenclature of each Rho GTPase is so confusing. Please follow the previous name: EHI_012240 as EhRacD and EhRho1 as EHI_029020, not EHI_013260.

**Response:** We apologise for the confusion. We understand that following the previous nomenclature would have been very much convenient, but the previous nomenclature is solely based on sequence similarity with the human Rho GTPase members (Lohia and Samuelson 1996). This method would identify different subfamilies of Rho GTPases including Rho subfamily and Rac subfamily members. In contrast, we have selected the Rho members based on the presence Rho specific insert helix (Zong et al; 2001) in addition to the presence of a classical G domain as predicted by the primary sequence (Van Aelst and D’Souza-Schorey 1997; Rossman, Der, and Sondek 2005). This selection criterion excluded some of the earlier designated amoebic Rho GTPases from the Rho family. For example: EhRho1 (EHI_029020) is not selected for the study as it lacks Rho specific insert helix. Similar nomenclature has been followed in a recent publication from our group (Narooka et al; 2022). However, for convenience, we have now added a table containing the old and new nomenclature along with the gene ID for naming the proteins used in this study (Table S3).

From line 321, it is shown that EhGEF2 activates EhRacG as a preferential substrate among the Rho family GTPases. To emphasize the importance of EhGEF2 for EhRho5, it is preferable to show how much the activation of EhRho5 by EhGEF2 occurs in vivo or in the presence of EhRacG.

Response: We thank the reviewer for suggesting this important experiment because it helps in understanding the substrate specificity inside the cells. Unfortunately, in spite of our serious effort we were not able to generate any specific antibody against endogenous EhRho5, thus could not measure the activation status of the GTPase in the EhGEF2 depleted or overexpressing condition.

However, we attempted to measure nucleotide exchange activity of EhGEF2 on EhRacG using *in vitro* GEF assay and compared the same with that on EhRho5 (EhRacD). In consistency with an earlier study (González De la Rosa et al. 2007) we observed that EhGEF2 also acts as a GEF for EhRacG, with a catalytic efficiency of 0.7 M^-1^ S^-1^, comparable to that observed for EhRho5. (Line 328-332).

Although the *in vitro* catalytic efficiency provides mechanistic information about the substrate specificity of a GEF at the molecular level, the cellular activity of a GEF on a GTPase also largely relies on the relative sub-cellular localization of the GEF-substrate pair. While under normal growth condition, EhRacG, similar to EhRho5 has been shown to be predominantly localised in cytosol, its localization upon LPA stimulation is yet to be studied (Guillén, Boquet, and Sansonetti 1998). Therefore, the present information seems inadequate to establish whether EhGEF2 has promiscuous activity on both the *in vitro* GTPase substrates. Of note, promiscuity of guanine exchange factors (GEF) or GTPase activating proteins (GAP) are not rare in the literature (Abe et al. 2000)(Han et al. 1997). (Line.418-426)

Also, overexpression of the GEF led to the formation of numerous macropinocytic cups at the surface, which became more pronounced post LPA stimulation (Fig.5H, Fig.S5H-I). The fact that no such phenotypic alterations were observed for HA-EhRho5 trophozoites, indicated the existence of more complex regulation of macropinocytosis, involving additional substrates GTPases for EhGEF2. In this context, EhRacG may also be a potential candidate to be investigated in future studies. (Line 493-496)

We have now discussed this possibility in the revised manuscript.

As stated in the introduction, EhRho5 is found in the phagosome proteome. How do authors find the main role of EhRho5 is in macropinocytosis?

Response: We appreciate the reviewer’s query and have added the clarification for choosing to investigate the role of EhRho5 in amoebic macropinocytosis to the revised manuscript. An earlier study has shown the association of EhRho5 with phagosomes, alongside EhRacA, EhRacC and EhRacG (Okada et al. 2006). Macropinocytosis and phagocytosis, collectively known as heterophagy are required for internalisation of extracellular material for nutrient acquisition and is required for amoebic sustenance (Junemann et al. 2016). While transiting from intestinal site of infection to the extraintestinal secondary infection sites, such as in case of hepatic amoebiasis, the amoebic trophozoites enter the bloodstream, where it experiences various factors from the host environment and may utilize them for nutrient acquisition and pathogenesis. LPA and PDGF are among such few host factors, produced by platelets which are present in the bloodstream (Czarkowska-Paczek, Bartlomiejczyk, and Przybylski 2006; Sugiura et al. 2002)(Line 78-88). Despite a lot of differences in the overall mechanism including the nature of stimuli and size of the compartments, initiation of macropinocytic and phagocytic membrane protrusions involves an overlapping set of regulatory and cytoskeletal proteins. While phagocytosis has been explored quite extensively in the amoebic trophozoites (Babuta et al. 2015; Verma and Datta 2017; Bharadwaj et al. 2021; Mansuri, Bhattacharya, and Bhattacharya 2014; Verma, Nozaki, and Datta 2016), studies addressing the cellular and molecular mechanism underlying macropinocytosis are limited (Laughlin et al. 2004; S. K. Ghosh and Samuelson 1997; Meza and Clarke 2004). This prompted us to sought to investigate the signalling responsible for macropinocytosis. (Line44-48)

What is the definition of micropinocytosis and pinocytosis? The authors used dextran uptake as a macropinocytosis model. However, the efficiency of the dextran uptake is explained as “pinocytic efficiency”. Pinocytosis is generally used for selective uptake mediated by clathrin and/or caveolae and the size of internalized vesicle is small (<150 nm). In contrast, macropinocytosis is non-selective and receptor independent process generate larger vesicle (0.2~5 μm). It has better to use “macropinocytic efficiency”. Related to this, images to show macropinosomes in figures 3 and 5 are poor. Most of the trophozoites did not acquire dextran and sometimes arrows indicated extracellular aggregate. Please show clear images.

Response: We are extremely sorry for the mistake in denoting macropinocytic efficiency. Necessary modifications have been made and incorporated in the revised manuscript. We thank the reviewer for pointing it out.

We sincerely apologise for not adding the most representative images showing macropinocytosis in the amoebic trophozoites and have therefore replaced the images with better quality images in Fig.3A

In the current study, we carried out a confocal microscopy based assay for studying macropinocytosis in the amoebic trophozoites, which was also used in earlier studies (Castellanos-Castro et al. 2016). As part of the protocol, we had performed a minimum of 3 washing steps post incubation with Dextran to remove the extracellular dextran from the depression slide. However, in a few instances, like in Fig. 5 D. wild type trophozoites stimulated with LPA showed extracellular dextran. For better clarity, we have now shown the cell boundary in the images showing dextran uptake and the arrowheads represent the internalised dextran. We would also like to point out since we have used confocal microscopy to image the macropinocytic events, the chance of counting a dextran punctae as extra/intra cellular entity would be limited by the optical resolution which is true for any confocal based imaging technique.

We agree with the reviewer that the uptake of dextran by the trophozoites are not uniform ranging from trophozoites with ‘0’ macropinosomes to that containing ‘n’ macropinosomes. We believe that this variation is naturally expected from a population of individual organisms. While quantifying the efficiency of macropinocytosis in gene depleted or control trophozoites, the entire population was taken into account.

Authors showed EhRho5 preferentially bound to RBD, not to PBD. Please discuss this point based on the amino acid sequence of EhRho5.

Response: We sincerely thank the reviewer for bringing it up. We would like to add that though His-EhRho5 binds to both RBD and PBD as concluded from our *in vitro* recombinant EhRho5, GST-RBD, GST-PBD pull down assay (fig 2A), when assayed for the nucleotide dependency of binding, we observed that it is only RBD which responses differentially to the GDP bound inactive EhRho5 from the GTP(GMPPNP) bound active EhRho5. Of note, by definition of a GTPase effector, which preferentially recognised the GTP bound activated form of a GTPase, we concluded that RBD is the genuine effector of EhRho5 (Line 172-178). Additionally, in HA-EhRho5 expressing trophozoites, the GTPase did not show any binding to GST-PBD further strengthening our conclusion that EhRho5 belongs to Rho sub family rather than the Rac sub family (Line 184-186). Accordingly, we have used RBD based pull down assay to measure the activation state of the GTPase under different condition, such as in the presence or the absence of LPA. This approach has been extensively employed in numerous studies including some on the amoebic trophozoites (Bharadwaj et al. 2021; Franco-Barraza et al. 2006).

It is known that the effector binding by Rho GTPases is generally attributed to Switch I region (YVPTVFDNY; EhRho5) and the ‘Rho insert’ region (EAMIRKLADENQK; EhRho5) (Rose et al. 2005; Mosaddeghzadeh and Ahmadian 2021). Among these two regions, while the switch I region is in more close vicinity to the nucleotide moiety through interaction with the Mg^2+^ ion via conserved threonine (Thr42; EhRho5). The Rho insert with helical secondary structure also shows conformational alteration upon GTP/GDP exchange during the activation of the GTPase. Of note, although the above regions are crucial for binding to the effector molecules and conserved among the different Rho family members, less conserved regions on the GTPase too determine effector binding (Rose et al. 2005; Mosaddeghzadeh and Ahmadian 2021). Thus, preferential binding to RBD by EhRho5 (Fig.2) may be attributed to the variable regions on the GTPase. (Line 396-409)

Localization of HA-EhRho5 and GFP-EhRho5 is significantly different. In the steady-state, HA-EhRho5 and GFP-EhRho5 are preferentially localized in the cytoplasm and on the membrane, respectively. The authors used HA-EhRho5 to show LPA stimulated translocation to membrane and GFP-EhRho5 to show the opposite effect by Wortmannin. It is not fair. It is necessary to examine the localization of endogenous EhRho5 by using a specific antibody detecting endogenous protein.

Response: We are grateful to the reviewer for pointing out the ambiguity. This indeed led to further reanalysis, performing new sets of experiments and strengthen our conclusions.

We agree that the sub cellular localization of a protein could be best determined by using a specific antibody which works for both immuno-blotting and immuno-cyto chemistry. Unfortunately, in spite of repeated efforts, we were not able to generate an antibody specific for detecting endogenous EhRho5. Therefore, we carried out our entire study using HA or GFP tag EhRho5 expressed at a near endogenous level using amoebic cysteine synthase promoter (Nakada-Tsukui et al. 2009). Additionally, to avoid any potential cell line-based artifacts, we have now generated a new set of cell lines expressing HA-EhRho5 or GFP-EhRho5 and investigated the effect of LPA on sub-cellular localization of the GTPase (Fig.S1D-E; Fig.S2G-I)

Analysis of the existing and new data sets of confocal microscopy-based images revealed that although, GFP-EhRho5 showed more membrane localization in the LPA untreated amoeba compared to that for HA-EhRho5, the response to LPA and its extent was equally evident for both (Sup Fig.1 and Sup Fig.2). We have also provided the biochemical evidence for LPA mediated translocation of EhRho5 (Fig1F), which represents a large population of amoebic trophozoites, thus providing a more generalised conclusion.

Further, to address the reviewer’s concern, we have now also investigated the involvement of PIK signalling in LPA mediated translocation of HA-EhRho5 using wortmannin based inhibition experiments in HA-EhRho5 expressing amoebic trophozoites. We observed that wortmannin treatment abrogates the translocation of HA-EhRho5 (Fig.4A-B) thus corroborating with our earlier observation from FRAP based measurements in GFP-EhRho5 expressing trophozoites (Fig.4A-B).

It is unclear what molecule is responsible for tethering EhRho5 on the plasma membrane shown by biotinylation. Please include this point in the discussion.

Response: We are thankful to the reviewer for pointing out the ambiguity. Rho GTPases like the most of the other Ras super family of Small GTPases tether to the inner leaflet of plasma membrane by the virtue of isoprenylation on the C-terminally located conserved CAAX motif (Michaelson et al. 2001). Therefore, to capture the membrane associated population of EhRho5, we used surface biotinylation approach, where Hgl, an integral membrane *protein of Entamoeba histolytica* served as a positive control for the biotinylation reaction. We used NP40, a mild detergent to avoid complete dissolution of the membrane (Line 134-139). For cell lysis we used NP40, a mild detergent to avoid complete dissolution of the plasma membrane (Buffer: 50 mM Tris-Cl pH 7.5, 150 mM NaCl, 1mM DTT and 1% NP-40 in the presence of protease inhibitor mixture-100 µM leupeptin, 10 µM pepstatin A, 0.3 µM aprotinin, 1 µM PMSF, and 10 µM E-64). Partial integrity of the lipid molecules in the membrane fragments thus generated, kept the integral and the tethered peripheral proteins associated. The lysate was incubated with NeutrAvidin beads for 30 mins at room temperature. The beads were washed with 1X PBS and the biotin labelled membrane fraction was eluted from the beads followed by Western blotting. (Line665-672)

Specificity of expression gene silencing is unclear. In EhRho5 and EhGEF2 silenced strain, please evaluate the level of another Rho and RhoGEF especially share a high identity. Adding to this, please explain more about the method for silencing. pKT3M vector is the name of the vector to express a myc-tagged protein in E. histolytica. Change the name of this vector appropriately. Include the information of what trigger sequence is in the plasmid. Does “WT control” means mock vector transformant or untransfected trophozoites?

Response: We are thankful to the reviewer for critically going through the methodology and the associated suggestion. We have now evaluated the expression levels of EhRacG (~55% identity with EhRho5) and EhGEF1 (~25% identity with EhGEF2) in EhRho5 and EhGEF2 depleted trophozoites, respectively (Sup Fig.3A and Sup Fig.5G). We have also elaborated on the methodology followed for gene silencing and accordingly modified materials and methods (Line 217-220) section.

We agree that pKT3M is the more common nomenclature for a myc tagged expression vector (https://doi.org/10.1074/jbc.M403987200), Dr. Upinder Singh’s group have used the same backbone devoid of myc to perform trigger induced gene silencing. We have used 4T trigger sequence (Morf et al. 2013) to perform gene silencing. The name of the vector has been modified appropriately in the revised manuscript.

The depletion of EhGEF2 and EhRho5 were compared with untransfected trophozoites. Also, for better clarity, we have now replaced ‘WT control’ with Untransfected trophozoites.

Please add more explanation for the FRAP assay. It is unclear what tau1 and tau2 mean. Also, please discuss whether this phenotype is general characteristics of Rho or EhRho5 specific.

**Response:** As suggested by the reviewer, we have now added further elaboration of the FRAP assay to the method section as well as whenever necessary, in the result section. We have also discussed the physical meaning of τ1 and τ2 and its possible physiological significances, in the discussion (Line458-462) section of the revised manuscript.

Authors claimed that EhGEF2 is the EhoGEF for EhRho5. This hypothesis comes from a precedence study. There are over 60 of RhoGEFs in E. histolytica. Please add discussion to explain the adequacy of this hypothesis. Also, to establish the PI3K-EhGEF2-EhRho5 axis, EhGEF2 should recognize 3-phosphorylated phosphatidylinositol, as discussed in the discussion and shown in Figure 6. In ref #54, the authors of the paper showed that recombinant EhGEF2 bound to phosphatidic acid but not the other phosphoinositides examined, such as PI(3)P, PIP2, and PIP3. Please explain the hypothesis include this result.

Response: As rightly pointed out by the reviewer, hypothesising a potential GEF from a list of 60 putative GEFs for a given substrate Rho GTPase is indeed a daunting task. In particular, in the absence of adequate number of studies on the amoebic GEFs, it was very much challenging to narrow down on the possible GEF candidates for EhRho5. We must accept that it is complete literature mining which has helped us on this occasion to find the reports on EhFP4 (Nakada-Tsukui et al. 2009) and EhGEF2 (González De la Rosa et al. 2007), which eventually led to inclusion of these two candidates for our study.

From our study, we have hypothesised that PI3K-EhGEF2-EhRho5 axis contribute to LPA stimulated macropinocytosis in the amoebic trophozoites. We have already shown (Fig.5) that LPA treatment causes translocation of HA-EhGEF2 from cytosol to membrane periphery. To prove the existence of PI3K-EhGEF2-EhRho5 axis, we first decided to show that translocation of EhGEF2 upon LPA stimulation could respond to the inhibition of PI3K activity. To probe the involvement of PI3K in translocation of EhGEF2, we used wortmannin based inhibition of PI3K. Serum starved HA-EhGEF2 expressing trophozoites were treated with wortmannin post LPA stimulation and fixed with paraformaldehyde. Cells were then processed for immunofluorescence and images were acquired using confocal microscopy. Inhibition of PI3K led to significant reduction in LPA mediated translocation of HA-EhGEF2 to the cell surface periphery by 52.6%, compared to that observed in the absence of any inhibition (Fig5).

The above results confirmed the involvement of PI3K activity in EhGEF2 translocation and prompted us to hypothesise that EhGEF2 should recognise a phosphorylated form of membrane PI, including phosphrylated phosphatidylinositols as suggested by the reviewer. Therefore, we next sought to identify the lipid binding specificity of EhGEF2. Recombinant His-EhGEF2 protein was employed to determine lipid binding specificity of the GEF in a lipid overlay assay. In contrast to the earlier reported study (González De la Rosa et al. 2007), the results from our study (Fig5, Sup. Fig.5) clearly demonstrated that EhGEF2 could bind strongly to PI(3)P, PI(4)P, PI(5)P and phosphatidylserine, along with weak binding to PtdIns(3,4)P2, PtdIns(3,5)P2, PtdIns(4,5)P2 and PtdIns(3,4,5)P3. In addition, EhGEF2 also binds to PA with variable binding capacity over different biological replicates (Line 352-369). It is known that the generation of PI(3)P , PI(3,4)P_2,_ PI(3,4,5)P_3_ and PI(4)P is governed by wortmannin sensitive PI3K and PI4K family of lipid kinases (Nakada-Tsukui et al. 2019). In *Entamoeba histolytica*, while PtdIns(3,4,5)P3 localized on the extended pseudopodia, and phagocytic cups (Byekova et al. 2010), and PtdIns3P on the phagocytic cups (Powell et al. 2005), and internal vesicles, PI(4)P and PI(3,4)P_2_ binding proteins were shown to reside on the plasma membrane (Morf et al. 2013)(Somlata, Nakada-Tsukui, and Nozaki 2017). Together our data suggests that membrane association of EhGEF2 is attributable to wortmannin sensitive signalling of different phosphorylated forms of phosphatidylinositol. (Line439-449)

The above sets of results and appropriate discussion have now been incorporated in the revised manuscript.

**Part III – Minor Issues: Editorial and Data Presentation Modifications**
Please use this section for editorial suggestions as well as relatively minor modifications of existing data that would enhance clarity.

Reviewer #1: 1. Line 88: The authors mention that the overexpressor is made using an “endogenous amoebic promoter.” This is misleading. Endogenous implies that there was an integration event into a chromosome so that expression is driven from a natural promoter. But, as far as this reviewer can tell, the overexpression plasmids remain episomal. The authors should clarify this point.

We sincerely apologise for the mistake and the inconvenience caused. We generated transgenic trophozoites by episomal overexpression of HA epitope tagged EhRho5 under amoebic cysteine synthase promoter to obtain a near endogenous level of expression (Nakada-Tsukui et al. 2009). The clarification has been incorporated now in the result section. (Line 111-113)

2. Line 105: To confirm plasma membrane localization of EhRho5 after stimulation, the authors stimulate cells, biotinylate the cell surface, and then purify labeled components with avidin. The authors show that EhRho5 is enriched in this purified fraction. In general, Rho proteins would not appear on the exoplasmic face of cells. Thus, they would not normally be exposed to be biotinylated. Therefore, how do the authors think the enrichment is happening? Is it because EhRho5 is interacting with another protein that would be exposed on the cell surface? The authors should discuss this point.

We appreciate the query raised by the reviewer. Rho GTPases like the most of the other Ras super family of Small GTPases tether to the inner leaflet of plasma membrane by the virtue of isoprenylation on the C-terminally located conserved CAAX motif (Michaelson et al. 2001). Therefore, to capture the membrane associated population of EhRho5, we used surface biotinylation approach, where Hgl, an integral membrane *protein of Entamoeba histolytica* served as a positive control for the biotinylation reaction. We used NP40, a mild detergent to avoid complete dissolution of the membrane (Line 134-139). For cell lysis we used NP40, a mild detergent to avoid complete dissolution of the plasma membrane (Buffer: 50 mM Tris-Cl pH 7.5, 150 mM NaCl, 1mM DTT and 1% NP-40 in the presence of protease inhibitor mixture-100 µM leupeptin, 10 µM pepstatin A, 0.3 µM aprotinin, 1 µM PMSF, and 10 µM E-64). Partial integrity of the lipid molecules in the membrane fragments thus generated, kept the integral and the tethered peripheral proteins associated. The lysate was incubated with NeutrAvidin beads for 30 mins at room temperature. The beads were washed with 1X PBS and the biotin labelled membrane fraction was eluted from the beads followed by Western blotting. (Line665-672). We have answered similar query raised by #reviewer2.

3. It is not clear what is gained by showing the microscopy images in Figure 3. For example, there isn’t any difference between -LPA and +LPA in 3A. There are minimal differences between cell lines in the TR-Dextran panels in 3C and 3E. Even though the panel for TR-Dextran for the HA-EhRho5-CA shows two endosomes (vs one endosome for the other cell lines), the authors are only showing one cell and not a field of cells. The quantification (3B,D,F,H) and the accompanying videos are sufficient support for the conclusions.

We sincerely apologise for not adding the most representative images showing macropinocytosis in the amoebic trophozoites and have therefore replaced the images with better quality images in Fig.3A.

In the current study, we carried out a confocal microscopy based assay for studying macropinocytosis in the amoebic trophozoites, which was also used in earlier studies (Castellanos-Castro et al. 2016). We would also like to point out that we have used corrected total cell fluorescence (CTCF) instead of comparing number of macropinosomes to measure the dextran uptake, which being an intensity-based analysis, provides more precise quantification of the process. Details of the method followed for the analysis are provided in the methods section. However, for representation purpose and to show raw experimental data, we have kept the rest of the figure unaltered.

4. The technical abstract does would not stand alone in a database such as PubMed. There is no mention of the pathogen until the last line. The whole genus name is not mentioned. There is no information about the importance of this organism. Readers outside the field, who arrive at this abstract in a database, would not fully understand the importance of the study.

We are thankful to the reviewer for these suggestions. We have now incorporated the importance of *E. histolytica* and physiological relevance of our study in the revised manuscript. (Line 9-11;19-21)

5. The authors mention in Line 43 of the Introduction that E. histolytica can take up 15% of cell volume. In what time frame (1 hour? 1 day?)? The information doesn’t mean much unless a time frame is given.

We have incorporated the complete information in the revised manuscript. *E. histolytica* is known to take up fluid corresponding to ~15% of its cell volume in 2hrs, showing its high ‘drinking’ capacity (Cohn, Scott, and Diseases 1984).(Line48-50)

6. In several instances in the Introduction and Discussion, the authors call Dictyostelium a “homologue” of E. histolytica. Organisms cannot be homologues of one another. They can be genetically related.

We apologise for the incorrect notations for the organism. Modifications have been incorporated in the revised manuscript.

7. Line 187: The authors state the “EhRho5 trophozoites showed no significant change in pinocytic efficiency…” Do the authors mean HA-EhRho5 trophozoites?

We thank the reviewer for pointing out our negligence. We have corrected the modified the text in the revised manuscript.

8. Throughout the Methods the authors use % as a unit (e.g., heat inactive serum, paraformaldehyde, triton-x-100, fbs, skim milk etc.). The reviewer assumes this is % (v/v), but the authors should include the complete unit.

We have incorporated the suggested modifications in the materials and methods in the revised manuscript.

9. Line 430: The authors mention using tetracycline to induce expression. It’s not clear which of the Entamoeba expression vectors, used in this study, are tet-inducible and which ones are not tet-inducible. When did the authors use tetracycline to induce expression and what proteins? There is no mention of tetracycline induction when describing the experiments in the Results. This information is important because it helps to reveal if the correct controls are used (e.g., irrelevant tet-inducible protein or empty plasmid).

We thank the reviewer for pointing out the ambiguity in the text. We would like to state that pTExGFP-EhRho5 is a tetracycline inducible plasmid. Thus, the experiments with GFP-EhRho5 indicate the tet-induced expression of EhRho5. The Western blot has been incorporated in the Fig.S2 for tet-induced expression. Of note, the experiments always demanded the expression of EhRho5, hence the uninduced cells have only been utilized to check the expression. These details have now been incorporated in the materials and methods section. (Line 191-193; 551-553)

10. In the Methods, the Western blotting protocol is not adequately described. For example, how long did the authors incubate with primary and secondary antibody? How many washes? How were the blots developed?

We apologise for inadequate description of Western blotting. Details of primary and secondary antibodies, incubation time, washes, detection system has now been incorporated in the revised manuscript. (Line 561-565)

11. The Methods are lacking the information on how cells were treated with LPA, PDGF, or wortmannin. Although the information is in the figure legends it should be included in the Methods.

We are thankful to the reviewer for the suggestion. It has enriched the section. We have modified the materials and methods section on the treatments, duration, etc. However, we would like to keep the information in the figure legends also, as it makes easier to understand the figure. (Line 567-571)

12. In the Methods and Acknowledgements, the authors thank a number of individuals for reagents and helpful suggestions. However, the authors never present the affiliations of these other scientists. The authors should include this information.

We apologise for the lack of information. The affiliations have now been added in the acknowledgements.

Reviewer #2: In line 28, “dysentery, abscesses and diarrhoea” would be “dysentery, diarrhoea, and abscess formation”.

“dysentery, abscesses and diarrhoea” has now been modified to “dysentery, diarrhoea, and abscess formation”. (Line 31)

In line 42, refer some review article would be appropriate.

We have now referred to reviews as well in the revised text.

In line 61 and others, Dictyostellium should be Dictyostelium and also it is not be a “homologue” of E. histolytica. Please use appropriate explanation.

We have now corrected the spelling of Dictyostelium. We also have modified the text and refrained from mentioning Dictyostelium as homologue of *Entamoeba histolytica.*

In line 68 and others, please discuss the fact in E. histolytica and other model organisms in a clearly separated way. The references cited here are a mixture of the observations from this amoeba and other organisms. It is also applicable in the following text.

We apologise for the ambiguity. We have moved the relevant references adjacent to the information provided to avoid confusion. Although various studies have reported induction of macropinocytosis by growth factors via activation of small GTPases (Nakase et al. 2015; Thompson and Bielska 2019; West et al. 2000), studies demonstrating their role in *E. histolytica* are still limited (S. K. Ghosh and Samuelson 1997). (Line 69-70)

In line 88, please clarify what promoter used to express EhRho5.

We used amoebic cysteine synthase promoter to express EhRho5 in trophozoites (Line 111-114). We have now added the detail of the promoter used to express EhRho5 in the revised manuscript.
In line 93, to quantify the membranous localization of HA-EhRho5 in Fig. 1B, how to define the “membrane”? Please clarify.

We apologise for the ambiguity. We have now added the clarification in materials and methods section as a new method (Line 592-602). We defined the cell periphery as the plasma membrane, confirmed by colocalization with Hgl- Heavy subunit of cell surface Gal/GalNAc lectin, in *Entamoeba histolytica.*

In line 97, “Gal-Gal Lectin” should be “Gal/GalNAc specific lection”. Please confirm.

“Gal-Gal Lectin” has now been modified as “Gal/GalNAc lectin”.

In fig.1C, cross section is applicable only a part of the cell. Please show the section of entire cell.
We have now incorporated the image showing the pixel intensity plot across the entire cell.

In line108, Neutravidin is a name of the product with trademark. It should be written as NeutrAvidinTM.

We thank the reviewer for pointing out this important detail. However, since the journal  do not publish any copyright or trademark symbols, we were unable to modify it.

In line 130, please add an explanation for adequacy to use representative Rho and Rac binding domains to E. histolytica Rho.

We thank the reviewer for the suggestion. GST-RBD and GST-PBD have been frequently used as bait to look for the presence of the activated, Rho-GTP population in numerous studies (D. Ghosh et al. 2021; Dise et al. 2007) including some in *E. histolytica* (Arias-Romero et al. 2006; Bharadwaj et al. 2021).

In line 148, it is indicated as ‘Fig. 2B’, but the effect of LPA stimulation is indicated in ‘Fig. 2D’.
We apologise for the negligence in referring to the figure. Suggested modification has been made in the revised manuscript.

In Fig. 3C, please depict the cell morphology at the middle panel for EhExHA.

We have now depicted the cell morphology of EhExHA in Fig.2C.

In Fig. 3C-H, it is unclear whether authors evaluated the macropinocytosis efficiency in HA- and GFP- expressed amoebas or all amoebas in the field. Please clarify.

We apologise for the ambiguity. We have now added the information in the materials and methods section. Cells expressing the HA-tagged proteins were analysed for their dextran uptake, but in the depletion cell lines, all the cells were taken in account for assessing their dextran uptake efficiency. (Line 582-584)

In line 222, it has better to refer original article to show PI3K inhibition by wortmannin.

We have now referred to original article related to the study.

In line 258, please spell out “PIP3”.

We apologise for the using the abbreviated form while introducing it for the first time in the manuscript. We have modified in the revised manuscript.

In line 293, it has better to cite original article related to this topic.

We have now referred to the original article. (Line 345-346)

In line 400, “BIS33 medium” or “BI-S-33 media”? Please be consistent.

We are sorry for the inconsistency in the text. We have now made sure regarding the consistency in the text post modifications.

In line 430, which transfectants are controlled by a tetracycline-inducible system is not clear. Please clarify.

We have incorporated this detail now in the materials and methods section.

In line 439, please describe references of antibodies used in this study.

We have now added the suitable references of antibodies used in the study.

In line 440, secondary antibodies for Western blotting are conjugated with which Alexa Fluor dye? Please clarify. Also, please add detection method of the image.

We apologise for missing out the details in the materials and methods. Information regarding the secondary antibodies in Western blot and the name of the detection method has been incorporated in the revised manuscript.

In line 446, anti-HGL should be anti-Hgl. Also, in the Western blotting, there are two kinds for this antibody were used. Which one has used for this assay?

anti-HGL has now been modified to anti-Hgl. We have incorporated the information in revised manuscript in materials section (Line 738).

In line 449, please add provider for the Mowiol.

The product details have now been added in the revised manuscript.

In line 463, what is the BIS33 incomplete media? “S” in the BI-S-33 medium stands for “serum”. If the “incomplete” mean serum-free medium, it should explain as BI medium. Or clarify what is missing in the incomplete medium.

We are thankful to the reviewer for the critical analysis of the manuscript. We agree with the reviewer that in BI-S-33 medium, S stands for “serum”. The incomplete medium is devoid of serum, vitamin mix, added in materials and methods. When using incomplete media in the study, BI-S-33 has been modified to BI medium.

In line 471~, please clarify the logic and calculation of how to measure the migration speed.

The clarification and the method have now been modified and added in the revised manuscript.

Analysis was performed using algorithms of the Icy software, a free and open-source platform for bioimage analysis that provides multiple resources to visualize annotate and quantify bioimaging data (<http://icy.bioimageanalysis.org>). Briefly, approximative polygonal Regions of Interest (ROIs) were drawn manually on frame 0 around each amoeba to initialize the segmentation for the Active Contours plugin (AC), then through AC method, each ROI polygon deforms to spouse the boundary of the segmented amoeba. Then, we used “Track objects over time” in the AC plugin with a volume constraint and volume weight 0.1. Once these parameters were established the segmentation was launched over the full sequence of frames. Once the segmentation was completed other the movie, AC sends the data to the Track Manager (TM) platform of the Icy software. As the amoeba shape was automatically tracked over time, the centroids of the successive ROIs were concatenated into a cell track by TM. The resulting tracks are directly overlayed on the original sequence. The TM module in Icy contains many Track Processors as Motion Profiler for quantifying cell motility parameters and cell morphology, such as cell speed (mm/s) and roundness (%), respectively. The speed is calculated with the displacement of the centroids of the successive ROIs over time and the “Roundness” is a measure of the similarity of the ROI to a circle. (Line 617-633)

In line 482~, plasmid construction method for pGEX plasmids was missing.

The pGEX plasmids (GST-RBD and GST-PBD) were a kind gift from Prof. Richard Cerione (Cornell University), thus the constructs were directly used for expression and purification of the proteins. The information has been mentioned now in the revised manuscript in methods explaining recombinant protein purification (Line 635).

In line 491, the detail for affinity purification is missing. It should be clarified to understand the recombinant protein purification process shown in Fig. S2A~C and Fig. S5A and B.

Details of the affinity purification for Fig.S2A-C as well as Fig.S5A-B have been incorporated in the manuscript.

In line 514 and 849, the recovery time is indicated as τ1/2, but in other parts, such as line 160 and line 162, the recovery time is indicated as t1/2. Please confirm.

We are grateful to the reviewer for pointing out the ambiguity in notations of the time constants. The time constants have been modified to τ1/2 in the revised manuscript.

In line 502m, “western blotting” should be “Western blotting”. The same applied to the entire text.

“western blotting” has now been modified to “Western blotting”.

In line 506, “GFP-Rho5” should be “GFP-EhRho5”.

GFP-Rho5 has now been modified to GFP-EhRho5.

In line 525, 0.1μMole should be 0.1 μmole. Also, add references for GST-RBD/PBD constructs.

We highly appreciate the changes suggested by the reviewer. 0.1μMole has been modified to 0.1 μmole. We have incorporated the references for GST-RBD and PBD constructs in the results and materials and methods section.

In line 563, “anti-mouse His” should be “anti-His monoclonal antibody”.
In line 564, “HA antibody” should be an “anti-HA monoclonal antibody”. The same applied to the expression of all the other antibodies, too.

Appropriate changes have been incorporated in the revised manuscript.

In Fig.6, the LPA receptor is not discussed in the main text. Please include. It has been shown that PI3P is on the endomembrane not on the plasma membrane in E. histolytica.
In figure legends, please state what showed in each panel, then explain the brief experimental procedure.

We are thankful for the critical review and suggestion. Using confocal microscopy and biochemical approaches we established that LPA stimulates the translocation and activation of EhRho5 (Fig.1, Fig.2). LPA mediated signalling has been implicated to function via GPCRs, to elicit diverse biological responses across various mammalian cell lines (Moolenaar 1995). Till date, three distinct GPCRs have been reported to contribute to LPA signalling in different tissues (Sugiura et al. 2002). Moreover, RhoA and Rac activation has been linked with LPA signalling via LPA_1_ receptors encoded by *edg2* gene (Van Leeuwen et al. 2003). Although a GPCR has been reported which directs bacterial engulfment in response to LPS(Lipopolysaccharide) stimulation, LPA receptors are yet to be reported in *E. histolytica* (Brewer et al. 2013) (Line 388-395)

In Fig. S5E, the image of anti-actin stating should be in the lower panel, same as in Fig. S3A.

We are sorry for the negligence while making the panels. The correction has been made in the both the revised figures (Fig.S5G; Fig.S3A).

Abe, Karon, Kent L. Rossman, Betty Liu, Kimberly D. Ritola, Derek Chiang, Sharon L. Campbell, Keith Burridge, and Channing J. Der. 2000. “Vav2 Is an Activator of Cdc42, Rac1, and RhoA.” *Journal of Biological Chemistry* 275 (14): 10141–49. https://doi.org/10.1074/jbc.275.14.10141.

Aelst, Linda Van, and Crislyn D’Souza-Schorey. 1997. “Rho GTPases and Signaling Networks.” *Genes and Development* 11 (18): 2295–2322. https://doi.org/10.1101/gad.11.18.2295.

Arias-Romero, Luis Enrique, Ma de Jesús Almáraz-Barrera, Juan Daniel Díaz-Valencia, Arturo Rojo-Domínguez, Rosaura Hernandez-Rivas, and Miguel Vargas. 2006. “EhPAK2, a Novel P21-Activated Kinase, Is Required for Collagen Invasion and Capping in Entamoeba Histolytica.” *Molecular and Biochemical Parasitology* 149 (1): 17–26. https://doi.org/10.1016/j.molbiopara.2006.04.001.

Babuta, Mrigya, M. Shahid Mansuri, Sudha Bhattacharya, and Alok Bhattacharya. 2015. “The Entamoeba Histolytica, Arp2/3 Complex Is Recruited to Phagocytic Cups through an Atypical Kinase EhAK1.” *PLoS Pathogens* 11 (12): 1–27. https://doi.org/10.1371/journal.ppat.1005310.

Bharadwaj, Ravi, Ranjana Arya, M. Shahid mansuri, Sudha Bhattacharya, and Alok Bhattacharya. 2017. “EhRho1 Regulates Plasma Membrane Blebbing through PI3 Kinase in Entamoeba Histolytica.” *Cellular Microbiology* 19 (10): 1–18. https://doi.org/10.1111/cmi.12751.

Bharadwaj, Ravi, Tushar Kushwaha, Azhar Ahmad, Krishna K. Inampudi, Tomoyoshi Nozaki, and Somlata. 2021. *An Atypical EhGEF Regulates Phagocytosis in Entamoeba Histolytica through EhRho1*. *PLoS Pathogens*. Vol. 17. https://doi.org/10.1371/JOURNAL.PPAT.1010030.

Blazquez, Samantha, Ghislaine Guigon, Christian Weber, Sylvie Syan, Odile Sismeiro, Jean Yves Coppée, Elisabeth Labruyère, and Nancy Guillén. 2008. “Chemotaxis of Entamoeba Histolytica towards the Pro-Inflammatory Cytokine TNF Is Based on PI3K Signalling, Cytoskeleton Reorganization and the Galactose/N-Acetylgalactosamine Lectin Activity.” *Cellular Microbiology* 10 (8): 1676–86. https://doi.org/10.1111/j.1462-5822.2008.01158.x.

Blazquez, Samantha, Christophe Zimmer, Ghislaine Guigon, Jean Christophe Olivo-Marin, Nancy Guillén, and Elisabeth Labruyère. 2006. “Human Tumor Necrosis Factor Is a Chemoattractant for the Parasite Entamoeba Histolytica.” *Infection and Immunity* 74 (2): 1407–11. https://doi.org/10.1128/IAI.74.2.1407-1411.2006.

Brewer, Matthew T., Prince N. Agbedanu, Mostafa Zamanian, Tim A. Day, and Steve A. Carlson. 2013. “Evidence for a Bacterial Lipopolysaccharide-Recognizing G-Protein-Coupled Receptor in the Bacterial Engulfment by Entamoeba Histolytica.” *Eukaryotic Cell* 12 (11): 1433–38. https://doi.org/10.1128/EC.00150-13.

Buckley, Catherine. 2018. “A Tale of Two Dicties: Macropinocytosis and Phagocytosis in the Social Amoeba Dictyostelium Discoideum,” no. March. http://etheses.whiterose.ac.uk/20249/.

Byekova, Yevgeniya A., Rhonda R. Powell, Brenda H. Welter, and Lesly A. Temesvari. 2010. “Localization of Phosphatidylinositol (3,4,5)-Trisphosphate to Phagosomes in Entamoeba Histolytica Achieved Using Glutathione S-Transferase- and Green Fluorescent Protein-Tagged Lipid Biosensors.” *Infection and Immunity* 78 (1): 125–37. https://doi.org/10.1128/IAI.00719-09.

Castellanos-Castro, Silvia, Carlos M. Cerda-García-Rojas, Rosario Javier-Reyna, Jonnatan Pais-Morales, Bibiana Chávez-Munguía, and Esther Orozco. 2016. “Identification of the Phospholipid Lysobisphosphatidic Acid in the Protozoan Entamoeba Histolytica: An Active Molecule in Endocytosis.” *Biochemistry and Biophysics Reports* 5: 224–36. https://doi.org/10.1016/j.bbrep.2015.12.010.

Cohn, Z A, W A Scott, and Infectious Diseases. 1984. “IN E N T A M O E B A” 160 (September): 724–37.

Czarkowska-Paczek, B, I Bartlomiejczyk, and J Przybylski. 2006. “The Serum Levels of Growth Factors: PDGF, TGF-Beta and VEGF Are Increased after Strenuous Physical Exercise.” *Journal of Physiology and Pharmacology : An Official Journal of the Polish Physiological Society* 57 (2): 189–97.

Dise, Rebecca S., Mark R. Frey, Robert H. Whitehead, and D. Brent Polk. 2007. “Epidermal Growth Factor Stimulates Rac Activation through Src and Phosphatidylinositol 3-Kinase to Promote Colonic Epithelial Cell Migration.” *American Journal of Physiology - Gastrointestinal and Liver Physiology* 294 (1): 276–85. https://doi.org/10.1152/ajpgi.00340.2007.

Franco-Barraza, Janusz, Horacio Zamudio-Meza, Elizabeth Franco, M. Del Carmen Domínguez-Robles, Nicolás Villegas-Sepúlveda, and Isaura Meza. 2006. “Rho Signaling in Entamoeba Histolytica Modulates Actomyosin-Dependent Activities Stimulated during Invasive Behavior.” *Cell Motility and the Cytoskeleton* 63 (3): 117–31. https://doi.org/10.1002/cm.20105.

Ghosh, Dipanjana, Ankita Dutta, Anjali Kashyap, Neeraj Upmanyu, and Sunando Datta. 2021. “PLP2 Drives Collective Cell Migration via ZO-1-Mediated Cytoskeletal Remodeling at the Leading Edge in Human Colorectal Cancer Cells.” *Journal of Cell Science* 134 (18). https://doi.org/10.1242/jcs.253468.

Ghosh, Sudip K., and John Samuelson. 1997. “Involvement of P21(RacA), Phosphoinositide 3-Kinase, and Vacuolar ATPase in Phagocytosis of Bacteria and Erythrocytes by Entamoeba Histolytica: Suggestive Evidence for Coincidental Evolution of Amebic Invasiveness.” *Infection and Immunity* 65 (10): 4243–49. https://doi.org/10.1128/iai.65.10.4243-4249.1997.

González De la Rosa, Claudia Haydée, Luis Enrique Arias-Romero, Ma de Jesús Almaraz-Barrera, Rosaura Hernandez-Rivas, Alejandro Sosa-Peinado, Arturo Rojo-Domínguez, Martha Robles-Flores, and Miguel Vargas. 2007. “EhGEF2, a Dbl-RhoGEF from Entamoeba Histolytica Has Atypical Biochemical Properties and Participates in Essential Cellular Processes.” *Molecular and Biochemical Parasitology* 151 (1): 70–80. https://doi.org/10.1016/j.molbiopara.2006.10.007.

Guillén, N, P Boquet, and P Sansonetti. 1998. “The Small GTP-Binding Protein RacG Regulates Uroid Formation in the Protozoan Parasite Entamoeba Histolytica.” *Journal of Cell Science* 111 ( Pt 1 (June): 1729—1739. http://europepmc.org/abstract/MED/9601102.

Han, J, B Das, W Wei, L Van Aelst, R D Mosteller, R Khosravi-Far, J K Westwick, C J Der, and D Broek. 1997. “Lck Regulates Vav Activation of Members of the Rho Family of GTPases.” *Molecular and Cellular Biology* 17 (3): 1346–53. https://doi.org/10.1128/MCB.17.3.1346.

Huston, Christopher D., and Adam Sateriale. 2011. “A Sequential Model of Host Cell Killing and Phagocytosis by Entamoeba Histolytica.” *Journal of Parasitology Research* 2011. https://doi.org/10.1155/2011/926706.

Junemann, Alexander, Vedrana Filić, Moritz Winterhoff, Benjamin Nordholz, Christof Litschko, Helena Schwellenbach, Till Stephan, Igor Weber, and Jan Faix. 2016. “A Diaphanous-Related Formin Links Ras Signaling Directly to Actin Assembly in Macropinocytosis and Phagocytosis.” *Proceedings of the National Academy of Sciences of the United States of America* 113 (47): E7464–73. https://doi.org/10.1073/pnas.1611024113.

Laughlin, Richard C., Glen C. McGugan, Rhonda R. Powell, Brenda H. Welter, and Lesly A. Temesvari. 2004. “Involvement of Raft-like Plasma Membrane Domains of Entamoeba Histolytica in Pinocytosis and Adhesion.” *Infection and Immunity* 72 (9): 5349–57. https://doi.org/10.1128/IAI.72.9.5349-5357.2004.

Leeuwen, Frank N. Van, Cristina Olivo, Shula Grivell, Ben N.G. Giepmans, John G. Collard, and Wouter H. Moolenaar. 2003. “Rac Activation by Lysophosphatidic Acid LPA1 Receptors through the Guanine Nucleotide Exchange Factor Tiam1.” *Journal of Biological Chemistry* 278 (1): 400–406. https://doi.org/10.1074/jbc.M210151200.

Mansuri, M. Shahid, Sudha Bhattacharya, and Alok Bhattacharya. 2014. “A Novel Alpha Kinase EhAK1 Phosphorylates Actin and Regulates Phagocytosis in Entamoeba Histolytica.” *PLoS Pathogens* 10 (10). https://doi.org/10.1371/journal.ppat.1004411.

Meza, Isaura, and Margaret Clarke. 2004. “Dynamics of Endocytic Traffic of Entamoeba Histolytica Revealed by Confocal Microscopy and Flow Cytometry.” *Cell Motility and the Cytoskeleton* 59 (4): 215–26. https://doi.org/10.1002/cm.20038.

Michaelson, David, Joseph Silletti, Gretchen Murphy, Peter D’Eustachio, Mark Rush, and Mark R. Philips. 2001. “Differential Localization of Rho GTPases in Live Cells: Regulation by Hypervariable Regions and RhoGDI Binding.” *Journal of Cell Biology* 152 (1): 111–26. https://doi.org/10.1083/jcb.152.1.111.

Moolenaar, Wouter H. 1995. “Lysophosphatidic Acid Signalling.” *Current Opinion in Cell Biology* 7 (2): 203–10. https://doi.org/10.1016/0955-0674(95)80029-8.

Moolenaar, Wouter H., Onno Kranenburg, Friso R. Postma, and Gerben C.M. Zondag. 1997. “Lysophosphatidic Acid: G-Protein Signalling and Cellular Responses.” *Current Opinion in Cell Biology* 9 (2): 168–73. https://doi.org/10.1016/S0955-0674(97)80059-2.

Morf, Laura, Richard J. Pearson, Angelia S. Wang, and Upinder Singh. 2013. “Robust Gene Silencing Mediated by Antisense Small RNAs in the Pathogenic Protist Entamoeba Histolytica.” *Nucleic Acids Research* 41 (20): 9424–37. https://doi.org/10.1093/nar/gkt717.

Mosaddeghzadeh, Niloufar, and Mohammad Reza Ahmadian. 2021. “The Rho Family Gtpases: Mechanisms of Regulation and Signaling.” *Cells* 10 (7). https://doi.org/10.3390/cells10071831.

Nakada-Tsukui, Kumiko, Hiroyuki Okada, Biswa Nath Mitra, and Tomoyoshi Nozaki. 2009. “Phosphatidylinositol-Phosphates Mediate Cytoskeletal Reorganization during Phagocytosis via a Unique Modular Protein Consisting of RhoGEF/DH and FYVE Domains in the Parasitic Protozoon Entamoeba Histolytica.” *Cellular Microbiology* 11 (10): 1471–91. https://doi.org/10.1111/j.1462-5822.2009.01341.x.

Nakada-Tsukui, Kumiko, Natsuki Watanabe, Tomohiko Maehama, and Tomoyoshi Nozaki. 2019. “Phosphatidylinositol Kinases and Phosphatases in Entamoeba Histolytica.” *Frontiers in Cellular and Infection Microbiology* 9 (JUN): 1–36. https://doi.org/10.3389/fcimb.2019.00150.

Nakase, Ikuhiko, Nahoko Bailey Kobayashi, Tomoka Takatani-Nakase, and Tetsuhiko Yoshida. 2015. “Active Macropinocytosis Induction by Stimulation of Epidermal Growth Factor Receptor and Oncogenic Ras Expression Potentiates Cellular Uptake Efficacy of Exosomes.” *Scientific Reports* 5 (January): 1–14. https://doi.org/10.1038/srep10300.

Okada, Mami, Christopher D. Huston, Miho Oue, Barbara J. Mann, William A. Petri, Kiyoshi Kita, and Tomoyoshi Nozaki. 2006. “Kinetics and Strain Variation of Phagosome Proteins of Entamoeba Histolytica by Proteomic Analysis.” *Molecular and Biochemical Parasitology* 145 (2): 171–83. https://doi.org/10.1016/j.molbiopara.2005.10.001.

Powell, R. R., Welter, B. H., Hwu, R., Bowersox, B., Attaway, C., & Temesvari, L. A. (2006). Entamoeba histolytica: FYVE-finger domains, phosphatidylinositol 3-phosphate biosensors, associate with phagosomes but not fluid filled endosomes. *Experimental parasitology*, *112*(4), 221-231.

Ríos, Amelia, Verónica Ivonne Hernández-Ramírez, Mayra Moguel, Alma Iris Zárate Bahena, José Luis Rosales-Encina, Miguel Angel Vargas, and Patricia Talamás-Rohana. 2008. “Participation of Rho, ROCK-2, and GAP Activities during Actin Microfilament Rearrangements in Entamoeba Histolytica Induced by Fibronectin Signaling.” *Cell Biology International* 32 (8): 984–1000. https://doi.org/10.1016/j.cellbi.2008.04.016.

Rose, R., M. Weyand, M. Lammers, T. Ishizaki, M. R. Ahmadian, and A. Wittinghofer. 2005. “Structural and Mechanistic Insights into the Interaction between Rho and Mammalian Dia.” *Nature* 435 (7041): 513–18. https://doi.org/10.1038/nature03604.

Seastone, David J., Eunkyung Lee, John Bush, David Knecht, and James Cardelli. 1998. “Overexpression of a Novel Rho Family GTPase, RacC, Induces Unusual Actin-Based Structures and Positively Affects Phagocytosis in Dictyostelium Discoideum.” *Molecular Biology of the Cell* 9 (10): 2891–2904. https://doi.org/10.1091/mbc.9.10.2891.

Shrimal, Shiteshu, Sudha Bhattacharya, and Alok Bhattacharya. 2010. “Serum-Dependent Selective Expression of EhTMKB1-9, a Member of Entamoeba Histolytica B1 Family of Transmembrane Kinases.” *PLoS Pathogens* 6 (6). https://doi.org/10.1371/journal.ppat.1000929.

Somlata, Kumiko Nakada-Tsukui, and Tomoyoshi Nozaki. 2017. “AGC Family Kinase 1 Participates in Trogocytosis but Not in Phagocytosis in Entamoeba Histolytica.” *Nature Communications*. https://doi.org/10.1038/s41467-017-00199-y.

Sugiura, Takayuki, Shinji Nakane, Seishi Kishimoto, Keizo Waku, Yasuko Yoshioka, and Akira Tokumura. 2002. “Lysophosphatidic Acid, a Growth Factor-like Lipid, in the Saliva.” *Journal of Lipid Research* 43 (12): 2049–55. https://doi.org/10.1194/jlr.M200242-JLR200.

Thompson, Craig B., and Agata A. Bielska. 2019. “Growth Factors Stimulate Anabolic Metabolism by Directing Nutrient Uptake.” *Journal of Biological Chemistry* 294 (47): 17883–88. https://doi.org/10.1074/jbc.AW119.008146.

Verma, Kuldeep, and Sunando Datta. 2017. “The Monomeric GTPase Rab35 Regulates Phagocytic Cup Formation and Phagosomal Maturation in Entamoeba Histolytica.” *Journal of Biological Chemistry* 292 (12): 4960–75. https://doi.org/10.1074/jbc.M117.775007.

Verma, Kuldeep, Tomoyoshi Nozaki, and Sunando Datta. 2016. “Role of EhRab7A in Phagocytosis of Type 1 Fimbriated E. Coli by Entamoeba Histolytica.” *Molecular Microbiology* 102 (6): 1043–61. https://doi.org/10.1111/mmi.13533.

West, Michele A., Alan R. Prescott, Eeva Liisa Eskelinen, Anne J. Ridley, and Colin Watts. 2000. “Rac Is Required for Constitutive Macropinocytosis by Dendritic Cells but Does Not Control Its Downregulation.” *Current Biology* 10 (14): 839–48. https://doi.org/10.1016/S0960-9822(00)00595-9.
